# Hyperpolarized [1-^13^C]pyruvate cardiovascular magnetic resonance imaging identifies metabolic phenotypes in patients with heart failure

**DOI:** 10.1016/j.jocmr.2024.101095

**Published:** 2024-09-11

**Authors:** Steen Hylgaard Joergensen, Esben Soevsoe S. Hansen, Nikolaj Bøgh, Lotte Bonde Bertelsen, Rasmus Stilling Tougaard, Peter Bisgaard Staehr, Christoffer Laustsen, Henrik Wiggers

**Affiliations:** a The MR Research Centre, Department of Clinical Medicine, Aarhus University, Aarhus, Denmark; b Department of Clinical Medicine, Aarhus University, Aarhus, Denmark; c Department of Cardiology, North Denmark Regional Hospital, Hjoerring, Denmark; d Department of Cardiology, Aalborg University Hospital, Aalborg, Denmark; e Department of Cardiology, Aarhus University Hospital, Aarhus, Denmark

**Keywords:** Heart failure, Cardiac metabolism, Metabolic imaging, Hyperpolarized [1-^13^C]pyruvate magnetic resonance imaging

## Abstract

**Background:**

Hyperpolarized [1-^13^C]pyruvate cardiovascular magnetic resonance imaging (HP [1-^13^C]pyruvate CMR) visualizes key steps in myocardial metabolism. The present study aimed to examine patients with heart failure (HF) using HP [1-^13^C]pyruvate CMR.

**Methods:**

A cross-sectional study of patients with HF and healthy controls using HP [1-^13^C]pyruvate CMR. Metabolic imaging was obtained using a cardiac-gated spectral-spatial excitation with spiral read-out acquisition. The metabolite signal was analyzed for lactate, bicarbonate, and the alanine signal. Metabolite signal was normalized to the total carbon signal (TC). At the 1-year follow-up, echocardiography was performed in all patients and HP [1-^13^C]pyruvate MRI in two patients.

**Results:**

We included six patients with ischemic heart disease (IHD), six with dilated cardiomyopathy, and six healthy controls. In patients, left ventricular ejection fraction (LVEF) correlated with lactate/bicarbonate (r = −0.6, p = 0.03) and lactate/TC (r = −0.7, p = 0.01). In patients with LVEF <30%, lactate/TC was increased (p = 0.01) and bicarbonate/TC reduced (p = 0.03). Circumferential strain correlated with metabolite ratios: lactate/bicarbonate, r = 0.87 (p = 0.0002); lactate/TC, r = 0.85 (p = 0.0005); bicarbonate/TC, r = −0.82 (p = 0.001). In patients with IHD, a strong correlation was found between baseline metabolite ratios and the change in LVEF at follow-up: lactate/bicarbonate (p = 0.001), lactate/TC (p = 0.011), and bicarbonate/TC (p = 0.012).

**Conclusions:**

This study highlighted the ability of HP [1-^13^C]pyruvate CMR to detect changes in metabolism in HF. HP [1-^13^C]pyruvate CMR has the potential for metabolic phenotyping of patients with HF and for predicting treatment response.

**Trial registration:**

EUDRACT, 2018-003533-15. Registered December 4, 2018, https://www.clinicaltrialsregister.eu/ctr-search/search?query=2018-003533-15.

## Graphical abstract

**Figure:**
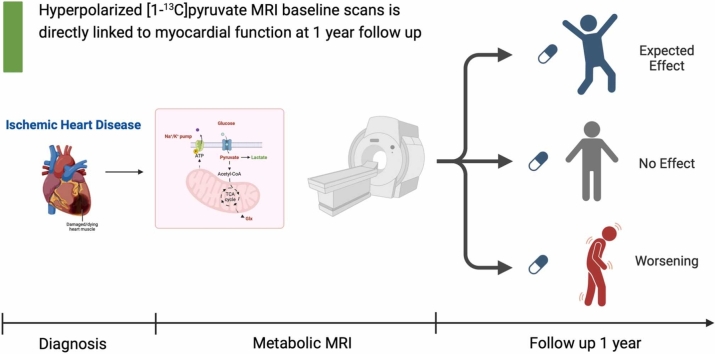

## 1 Introduction

Despite major progress in medical treatment of heart failure (HF), mortality and morbidity remain high [1], [2], [3], [4], [5]. HF is a heterogeneous disease. A comprehensive, non-invasive method for better phenotyping of patients with HF may therefore potentially improve prognostic stratification and personalize treatment selection [6], [7]. In recent years, abnormalities in myocardial metabolism have been shown to exacerbate and determine treatment response in HF [8], [9], [10], [11]. Hyperpolarized [1-^13^C]pyruvate cardiovascular magnetic resonance (HP [1-^13^C]pyruvate CMR) has emerged as a novel metabolic imaging method combining advanced metabolic and structural cardiovascular magnetic resonance (CMR) [12], [13], [14]. Hyperpolarization by the dissolution dynamic nuclear polarization technique transiently increases the magnetic resonance (MR) signal of [1-^13^C]pyruvate >10,000 times, enabling detection of intracellular myocardial lactate and bicarbonate production [15], [16], [17], [18]. Previously, HP [1-^13^C]pyruvate CMR has been applied in human studies of healthy hearts, diabetic hearts, and coronary artery disease [13], [16], [17], [19], [20]. To date, the method has not been applied in patients with HF. Hence, the overall objective of the present study was to implement HP [1-^13^C]pyruvate CMR in patients with HF for the first time. We aimed to (1) characterize myocardial pyruvate metabolism in patients with HF; (2) identify potential different metabolic phenotypes in patients with ischemic heart disease (IHD) and patients with dilated cardiomyopathy (DCM); (3) characterize regional differences in myocardial metabolism in HF; (4) characterize the association between changes in pyruvate metabolism and recovery of contractile myocardial function, and, finally, (5) compare pyruvate metabolism in patients with HF and a healthy control group. These aims were designed with a view toward attempting complete metabolic phenotyping of HF patients, which could potentially pave the way for truly personalized medicine in HF as well as unlock novel avenues of treatment for future research.

## 2 Materials and methods

### 2.1 Patients with heart failure and healthy controls

We screened 15 patients with HF. The inclusion criteria were (1) a diagnosis of HF according to guidelines [5]; (2) left ventricular ejection fraction (LVEF) <50%; (3) an estimated glomerular filtration rate >30 mL/min; (4) normal leukocyte and platelet counts, and (5) ability to give informed consent. The exclusion criteria were (1) a dose of more than 50% of the target dose for angiotensin-converting enzyme (ACE) inhibitor and beta blockers at inclusion; (2) any uncontrolled serious medical condition such as uncorrected heart valve disease or uncorrected hypertension; (3) insulin-dependent diabetes mellitus, and (4) intolerance to pyruvate. The patients had all been revascularized between 1 and 2 months before their baseline scan. Three were fully revascularized and three had chronic total occlusion of one coronary artery. None of the patients underwent any revascularization procedures in the period between baseline and follow-up. The control group consisted of six healthy volunteers from a previously published study [18]. The present study was conducted according to the Helsinki principles and approved by the National Committee on Health Research Ethics (2018-003533-15) and the Danish Medicines Agency (2019123690). Written informed consent was obtained from all patients before enrollment. All patients were recruited from the outpatient HF clinic at Aarhus University Hospital, Denmark. Controls were recruited through local advertisement. The study was performed at the Department of Cardiology and the MR Research Centre, Aarhus University Hospital, Aarhus, Denmark, between October 2019 and June 2022.

### 2.2 Study design

All patients and healthy controls underwent (1) a screening/inclusion visit, (2) a CMR study visit, and (3) a 1-year follow-up visit. Standard blood counts and metabolic profiles were obtained before and 7 days after the CMR study visit. Echocardiography was performed at inclusion, as previously described [10], and at 1-year follow-up. End diastolic and systolic volumes were measured by the Simpson biplane method to assess LVEF. Mitral inflow velocities (E and A) and mitral plane velocity in the lateral mitral annulus (e′) were measured. A GE Vivid E9 ultrasound machine (GE Healthcare, Chicago Illinois) was used for echocardiographic image acquisition and an EchoPAC 13 (GE-Vingmed Ultrasound, Horten, Norway) for analysis. Electrocardiogram was recorded.

### 2.3 Hyperpolarized magnetic resonance imaging

**Fig. 1:**
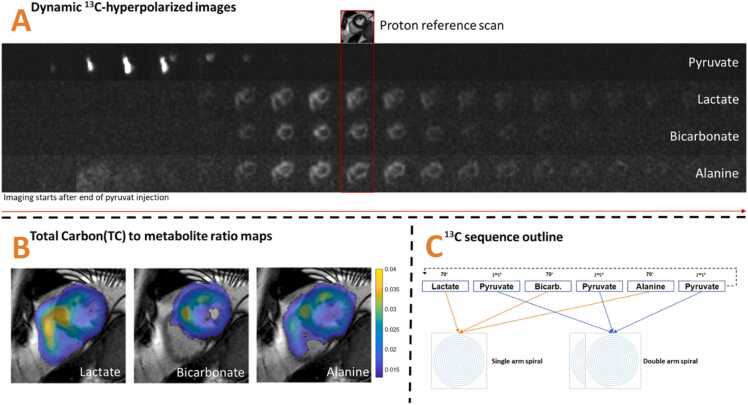
Illustration of HP [1-^13^C]pyruvate CMR imaging. (A) Dynamic hyperpolarized images of LV. (B) Overlay of metabolite ratios. (C) Sequence outline. *HP* hyperpolarized, *CMR* cardiovascular magnetic resonance, *LV* left ventricle

In the study of patients with HF and healthy controls alike [18], participants fasted for 8 to 15 h before HP [1-^13^C]pyruvate CMR. Cine CMR imaging was performed with the subjects in supine position. Participants ingested 75 g of oral glucose in 200 mL water 60 min before HP [1-^13^C]pyruvate CMR to maximize pyruvate dehydrogenase (PDH) flux [16]. Blood glucose concentrations were measured before and 1 h after glucose ingestion. A clinical polarizer (SPINlab, GE, Chicago Illinois) was used to polarize the sterile [1-^13^C]pyruvate. The release criteria have been described previously [16]. Briefly, proton images were acquired using an eight‐channel cardiac array receiver coil (GE Healthcare, Chicago Illinois) or the built-in body coil. For cine left ventricular function (cine LVF), we used balanced steady-state free precession. The sequence parameters were an echo time (TE) of 2.4 ms, repetition time of 5.1 ms, flip angle (FA) of 55°, acquisition matrix of 200 × 160, field of view (FOV) of 400 × 400 mm^2^, in-plane resolution 2 × 2.5 mm^2^, slice thickness 8 mm, recon matrix 512 × 512, and cardiac phases 30. Cine LVF was used to measure ejection fraction, strain feature tracking, and reference anatomy for the cine LVF [21], [22], [23]. Hyperpolarized [1‐^13^C]pyruvate CMR was undertaken with a transmit/receive Helmholtz loop-pair ^13^C coil (PulseTeq Limited, Surrey, UK) or a transmit clamshell coil with a 16-channel array receive coil (Rapid Biomedical GmbH, Rimpar, Germany). Transmit gain calibration was performed to adjust the radiofrequency (RF) power levels to the desired FAs for each subject, and ^13^C transmit power was calculated with a Bloch-Siegert method on a [^13^C]-bicarbonate phantom positioned in the coil sensitivity area and close to the imaging plane above the heart [24]. Transmit gain was measured to vary ∼0.2 dB across subjects. The imaging frequency for the hyperpolarized ^13^C imaging was calculated from the proton frequency obtained in the individual heart [25]. Hyperpolarized imaging was obtained using a cardiac-gated spectral-spatial excitation with spiral read-out acquisition. Patient data were obtained with these parameters: TE = 10 ms, read-out = 27 ms, FA = [pyruvate = 5°/5° [two spiral arms]; metabolites = 70°], FOV = 340 × 340 mm, matrix = [pyruvate = 90 × 90, metabolites = 40 × 40], native resolution = [8 × 8; 16 × 16] mm, and one short-axis slice with 30 mm slice thickness (Fig. 1C). The data for healthy subjects were obtained with the following parameters: TE = 10 ms, read-out = 34 ms, FA = [pyruvate;= 8°; metabolites = 90°], FOV = 400 × 400 mm, matrix = 30 × 30, native resolution = 13.3 × 13.3 mm, and one short-axis slice with 30 mm slice thickness [18], [26]. Hyperpolarized imaging from healthy subjects and patients was all reconstructed to an image matrix of 128 × 128. A total of 240 excitations produced 120 pyruvate images, leaving each metabolite with 40 images with a time resolution of three heartbeats.

### 2.4 Analyses of MRI and HP [1-^13^C]pyruvate data

Reconstruction of the ^13^C data and analysis of the digital imaging and communications in medicine images were performed in Segment version 3.1 R8215 (http://segment.heiberg.se) [23]. Regions of interest (ROIs) were analyzed by outlining the myocardium of the left ventricle (LV). The metabolite signal was analyzed at the mid-LV level as the mean signal from the myocardium and as the signal from the myocardium with ROIs in six mid-LV myocardial segments (anterior, antero-septal, antero-lateral, inferior, infero-septal, and infero-lateral). The area under the curve from the peak pyruvate signal and 10-time frames forward were calculated for each of the metabolites [27], [28]. We calculated the ratios of each individual metabolite to the total carbon signal (TC: lactate, bicarbonate, and alanine), and the balance between the glycolytic and oxidative weighted pyruvate metabolism (lactate/bicarbonate).

### 2.5 Statistical analysis

Normality of data was tested using the Shapiro-Wilk normality test. Analyses were performed in GraphPad Prism (GraphPad Software, San Diego, California). Results are presented as mean ± standard deviation (SD) or median [interquartile range, IQR], as appropriate. Unpaired t-tests were used to compare mean values between the two groups and a one-way analysis of variance (ANOVA) was used to compare segmental differences in myocardium. Two-way ANOVA was used to compare differences in myocardial segments and patients. The F-test was used to compare the variances of the two groups. Correlation was analyzed using the Pearson correlation coefficient and linear regression. Categorical variables were compared using Fisher’s exact test. We tested for outliers using the robust regression and outlier removal method. One outlier was identified in the healthy control group (S4). These data have been excluded from the analyses presented below. Two-tailed p-values were used. A p-value <0.05 was considered statistically significant.

## 3 Results

### 3.1 Patients

We screened 15 patients with stable HF and LVEF <50%. One withdrew consent and two were excluded because ACE inhibitor and beta blocker treatment had increased to >50% of the target dose in the time interval between inclusion and the first study visit. The final HF study population comprised 12 patients with HF who were studied with echocardiography and cine CMR. The historical control group of six healthy controls had undergone similar imaging [18]. The group of patients with HF counted six patients with IHD and six patients with DCM. Apart from their etiology, baseline variables in the two HF subgroups did not differ (Table 1). The healthy control group was significantly younger, had a lower glycated hemoglobin (HbA1c), and as intended had a normal LVEF and received no treatment for HF (Table 1). All participants were Caucasian.

**Table 1:** Baseline characteristics.

	HF (n = 12)	Controls (n = 6)	p-value	IHD (n = 6)	DCM (n = 6)	p-value
Age, y	63 ± 9	29 ± 7	<0.001*	64 ± 10	62 ± 9	0.6
Gender (male/female)	9/3	4/2	1.0	5/1	5/2	1.0
Duration of HF, mo	1 [0.5–6]	-	N/A	1 [0.5–1.3]	2.5 [1–5.3]	0.1
NYHA class I at enrollment, No. (%)	2 (17%)	-	N/A	0 (0%)	2 (33%)	0.5
NYHA class II at enrollment, No. (%)	10 (73%)	-	NA	6 (100%)	4 (67%)	0.5

*Laboratory parameters*						
Systolic BP, mmHg	123 [116–135]	122 [114–130]	0.7	126 [111–142]	121 [110–138]	0.8
Diastolic BP, mmHg	77 [72–80]	75 [69–81]	0.6	78 [68–82]	76 [73–78]	0.8
NT-proBNP, ng/L	963 [281–1932]	-	N/A	1345 [793–2455]	421 [212–1572]	0.3
NT-proBNP, ng/L at follow-up	-	-	N/A	599 [448–2347]	157 [121–349]	0.2
Creatinine, µmol/L	74 [62–88]	74 [69–78]	0.6	75 [70–91]	66 [56–92]	0.4
HbA1c, mmol/mol	40 [37–43]	34 [32–34]	0.002*	40 [38–43]	42 [36–48]	0.6
BMI, kg/m^2^	25 ± 3	23 ± 4	0.5	25 ± 3	24 ± 3	0.7
LVEF, %	31 ± 12	55 ± 4	<0.001*	31 ± 13	31 ± 13	1.0
LVEF, % at follow-up	-	-	N/A	44 ± 16	44 ± 11	0.2
E/e′	10 [7–11]	6 [6, 7]	0.07	10 [8–15]	11 [6–12]	0.8
LVEDV/BSA, mL/m^2^	85 ± 24	76 ± 13	0.4	87 ± 32	85 ± 17	0.8
LVESV/BSA, mL/m^2^	61 ± 24	33 ± 3	0.01*	63 ± 34	58 ± 16	0.9
LV mass/BSA, g/m^2^	76 ± 17	55 ± 2	0.01*	78 ± 19	71 ± 16	0.5
						
*Risk factors, No. (%)*						
Ischemic heart disease, No. (%)	6 (50%)	-	N/A	6 (100%)	-	N/A
Atrial fibrillation, No. (%)	2 (17%)	-	N/A	0 (0%)	2 (33%)	0.5
Left bundle branch block, No. (%)	2 (17%)	-	N/A	1 (17%)	1 (17%)	1.0
						
*Treatments*						
ß-blocker, No. (%)	10 (83%)	-	N/A	5 (83%)	5 (83%)	1.0
Mean achieved % of target dose	20%	-	N/A	20%	23%	0.8
ACE-I or ARB, No. (%)	12 (100%)	-	N/A	6 (100%)	6 (100%)	1.0
Mean achieved % of target dose	45%	-	N/A	39%	51%	0.3
Mineralocorticoid antagonist, No. (%)	2 (17%)	-	N/A	1 (17%)	1 (17%)	1.0
Mean achieved % of target dose	100%	-	N/A	100%	100%	1.0
Loop diuretics, No. (%)	7 (58%)	-	N/A	67%	50%	0.6
Mean dose (mg furosemide equivalents)	54	-	N/A	45	67	0.3

Data are presented as actual numbers (%), mean ± SD or median [IQR]

*ACE-I* angiotensin-converting enzyme inhibitor, *ARB* angiotensin receptor blocker, *BMI* body mass index, *BP* blood pressure, *BSA* body surface area, *DCM* dilated cardiomyopathy, *E/e′* early diastolic transmitral flow velocity to early diastolic mitral annular tissue velocity ratio, *HbA1c* glycated hemoglobin, *HF* heart failure, *IHD* ischemic heart disease, *IQR* interquartile range, *LV* left ventricle, *LVEDV* left ventricular end-diastolic volume, *LVEF* left ventricular ejection fraction, *LVESV* left ventricular end-systolic volume; *NT-proBNP* N-terminal pro-hormone brain natriuretic peptide, *NYHA* New York Heart Association, *SD* standard deviation

* P > 0.05

### 3.2 Specifications of injected hyperpolarized [1-^13^C]pyruvate

Twelve patients and six healthy controls had a successful HP [1-^13^C]pyruvate CMR at baseline. In two patients, this was repeated at the 1-year follow-up. Mean level of polarization was 36 ± 11%; mean pyruvate concentration was 248 ± 9 mM; mean pH was 7.7 ± 0.1; mean temperature was 36 ± 2°C; and mean residual electron paramagnetic agent concentration was 1.4 ± 0.7 µM. Immediately after injection, a hyperpolarized signal from [1-^13^C]pyruvate appeared in the lumen of the right ventricle and subsequently in the LV; and 2–3 s later, downstream metabolites (lactate, bicarbonate, and alanine) were detected in the myocardium of the LV (example shown in Fig. 1). No adverse events occurred. Time from dissolution to injection in patient was 80–100 s.

### 3.3 Findings by HP [1-^13^C]pyruvate CMR in patients with heart failure and reduced ejection fraction

**Fig. 2:**
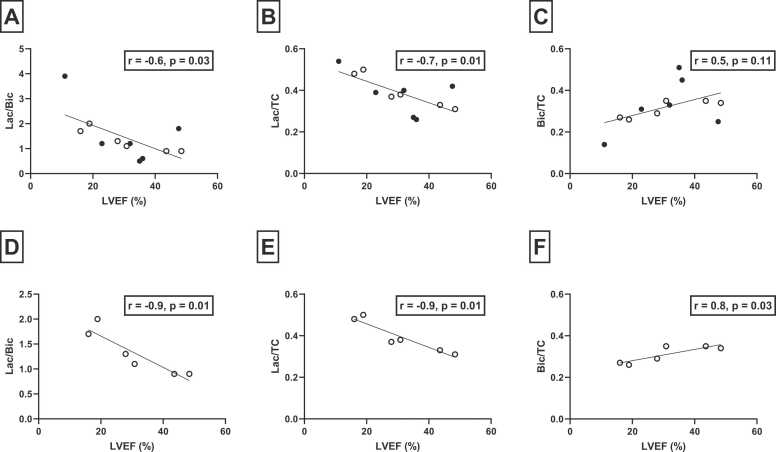
Correlation between LVEF and metabolite ratios for all patients with heart failure (A-C) and for IHD patients alone (D-F). о, IHD patients; •, DCM patients. *LVEF* left ventricular ejection fraction, *IHD* ischemic heart disease, *DCM* dilated cardiomyopathy

A negative correlation was observed between LVEF and lactate/bicarbonate (r = −0.6, p = 0.03) and lactate/TC (r = −0.7, p = 0.01) but not for bicarbonate/TC (r = 0.5, p = 0.11). Subgroup analyses showed that correlations between LVEF and metabolite ratios were highly significant in the IHD group; lactate/bicarbonate (r = −0.9, p = 0.01); lactate/TC (r = −0.9, p = 0.01); and bicarbonate/TC (r = 0.8, p = 0.03) (Fig. 2). In the DCM group, these correlations were similar, yet statistically insignificant. We found a statistically significant correlation between poor mid-LV circumferential strain values and metabolite ratios: lactate/bicarbonate, r = 0.87 (p = 0.0002); lactate/TC, r = 0.85 (p = 0.0005); bicarbonate/TC, r = −0.82 (p = 0.001). Furthermore, we found a non-statistically significant weak positive correlation between HbA1c and the lactate/bicarbonate ratio (r = 0.5, p = 0.08) (Supplementary Fig. 6), but no correlation between metabolite ratios and age, body mass index (BMI), LV mass, N-terminal pro-hormone brain natriuretic peptide (NT-proBNP), New York Heart Association (NYHA) class or dose of HF medication and etiology of HF (IHD versus DCM) (Supplementary Data).

### 3.4 HP [1-^13^C]pyruvate CMR in patients with heart failure and healthy controls

**Fig. 3:**
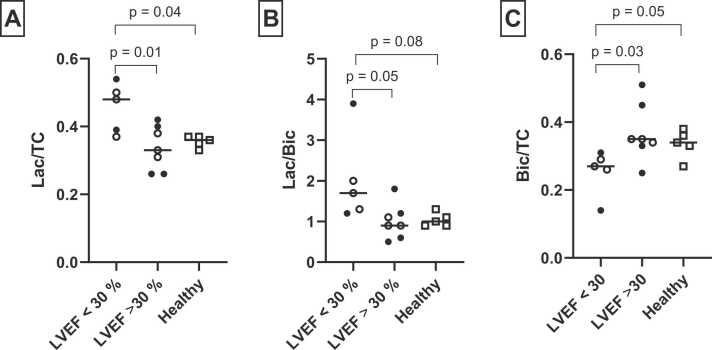
Comparison of metabolite ratios in patients with HF with LVEF <30%, LVEF ≥30%, and healthy controls. о, IHD patients; •, DCM patients; □, healthy controls. *HF* heart failure, *LVEF* left ventricular ejection fraction, *DCM* dilated cardiomyopathy, *TC* total carbon signal, *Lac* lactate, *Bic* bicarbonate

The lactate/bicarbonate ratio was 1.2 ± 0.5 in patients with HF versus 1.0 ± 0.2 in healthy controls (p = 0.5); the lactate/TC ratio, 0.39 ± 0.1 versus 0.36 ± 0.01 (p = 0.5), and the bicarbonate/TC ratio, 0.32 ± 0.1 versus 0.34 ± 0.04 (p = 0.7). When lactate/bicarbonate, lactate/TC, and bicarbonate/TC were plotted against LVEF for both patients with HF and healthy controls, it was evident that below an LVEF of 30%, the metabolite ratios changed (Supplementary Data). Lactate/TC was significantly higher in patients with an LVEF <30% than in those with LVEF ≥30% (p = 0.01) and in healthy controls (p = 0.04) (Fig. 3). A trend was observed toward a higher lactate/bicarbonate ratio in LVEF <30% than in LVEF ≥30% (p = 0.05) and in healthy controls (p = 0.08). Bicarbonate/TC was lower in LVEF <30% than in LVEF ≥30% (p = 0.03) but not significantly lower than in healthy controls (p = 0.05).

### 3.5 HP [1-^13^C]pyruvate CMR identifies increased metabolic variance in patients compared with healthy controls

**Fig. 4:**
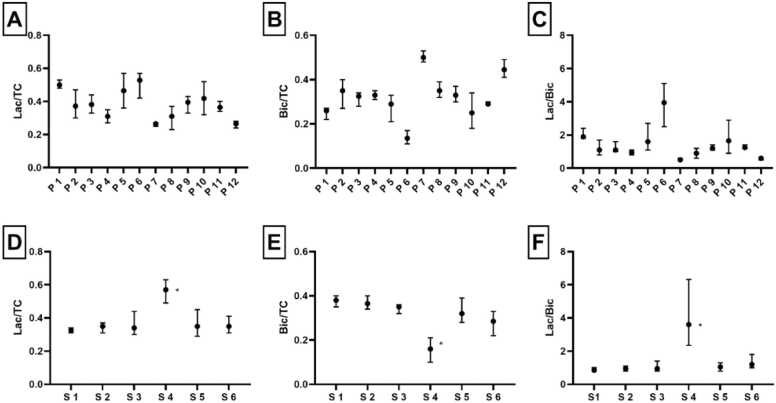
Above, each patient (P 1–12) is represented with mean myocardial Lac/TC (A), Bic/TC (B), and Lac/Bic (C). Below, each healthy subject (S 1–6) is represented similar to the patients (D-F). The whiskers show a range in the six segments. S4 was an outlier (*). *TC* total carbon signal, *Lac* lactate, *Bic* bicarbonate

**Fig. 5:**
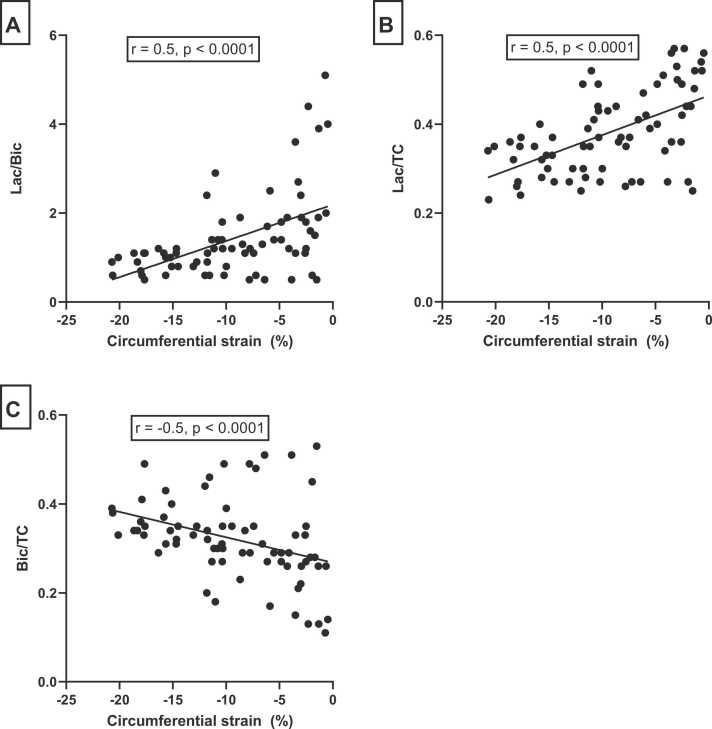
Correlation of circumferential strain and metabolite ratios for patients with heart failure (A-C). Metabolite ratios and strain values from each segment in every patient were pooled in the analysis. *TC* total carbon signal, *Lac* lactate, *Bic* bicarbonate

**Fig. 6:**
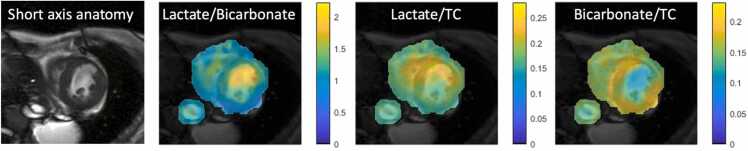
Illustration of metabolic imaging in a patient with HF and fully revascularized anterior transmural myocardial infarction 1 month before hyperpolarized MRI. Proton image shows thinning of the myocardium in the infarcted area. There is reduced bicarbonate signal in the infarcted area. *HF* heart failure, *TC* total carbon signal, *MRI* magnetic resonance imaging

There were significant differences in mean metabolite ratios between patients: lactate/TC (p < 0.005), bicarbonate/TC (p = 0.004), and lactate/bicarbonate (p = 0.0001) (Fig. 4 A-C). The mean levels of metabolite ratios did not differ significantly between subjects in the healthy control group (Fig. 4 D-F). Statistically significant differences in variances of metabolite ratios were seen between patients with HF and healthy controls: lactate/bicarbonate (p = 0.006) and lactate/TC (p = 0.002). However, for bicarbonate/TC, the difference in variance between the two groups was insignificant (p = 0.1) In patients with HF, segmental myocardial function was analyzed by circumferential strain analyses in the same six segments. We found a statistically significant correlation between poor circumferential strain values and lactate/bicarbonate ratio (r = 0.5, p = 0.0001); and similarly for lactate/TC ratio (r = 0.5, p = 0.0001) (Fig. 5). A statistically significant negative correlation was found between poor circumferential strain values and bicarbonate/TC ratio (r = −0.5, p = 0.0001). In Fig. 6, an example of regional differences in metabolite signal is illustrated in a patient with prior anterior transmural myocardial infarction.

### 3.6 HP [1-^13^C]pyruvate CMR at baseline and myocardial function at follow-up

**Fig. 7:**
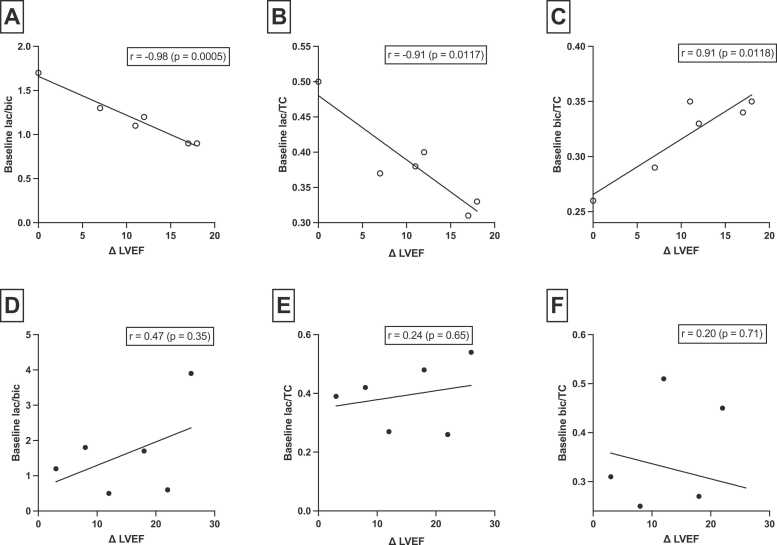
Correlation between baseline metabolite ratios and difference in LVEF from baseline to follow-up (Δ LVEF) in patients with HF. о, IHD patients; •, DCM patients. *LVEF* left ventricular ejection fraction, *HF* heart failure, *IHD* ischemic heart disease, *DCM* dilated cardiomyopathy, *TC* total carbon signal, *Lac* lactate, *Bic* bicarbonate

Echocardiography at baseline showed a mean LVEF of 31 ± 8% compared with a mean LVEF of 43 ± 10% at follow-up (p < 0.001). NYHA class at baseline versus at follow-up improved such that 2 patients were in NYHA I and 10 in NYHA II at baseline compared to 9 patients in NYHA I and 3 in NYHA II at follow-up (p = 0.01). Median NT-proBNP improved from 963 [281–1932] ng/L to 418 [138–622] ng/L (p = 0.14). Mean follow-up time was 13 ± 3 months. When patients were dichotomized at follow-up into non-recovered LVEF (<40%) and recovered LVEF (≥40%), no significant difference was found in mean baseline metabolite ratios between the two groups. However, subgroup analysis of the patients with IHD (Fig. 7) showed a significant correlation between baseline metabolite ratios and improvement in LVEF at follow-up (Δ LVEF): lactate/bicarbonate, r = −0.98, p = 0.0005; lactate/TC, r = −0.91, p = 0.0117; bicarbonate/TC, r = 0.91, p = 0.0118.

## 4 Discussion

The present study represents the results of the first investigation of HP [1-^13^C]pyruvate CMR in patients with HF. First, we observed increased lactate/bicarbonate and lactate/TC ratios in patients with lower LVEF and circumferential strain. Second, the considerable variability between patients suggests that HP [1-^13^C]pyruvate CMR may aid metabolic phenotyping of patients with HF. Third, in patients with IHD, increased lactate ratios and reduced bicarbonate ratios at baseline predicted poor LVEF improvement at follow-up.

### 4.1 Changes in lactate metabolism in patients with chronic heart failure of different etiologies

We found a strong correlation between high lactate signal and poor contractility in patients with HF and IHD, indicating altered lactate handling in this group. This is in line with previous findings in a porcine model of chronic infarction [29]. It has previously been debated whether the myocardial lactate signal following a pyruvate injection may be contaminated by lactate from the blood pool [13], [16], [30]. While this is true for non-localized MR spectroscopy, we believe that metabolic imaging, as seen in the present study, increases the likelihood of correctly defining whether lactate signal is of myocardial origin. Still, the detected changes in lactate signal may not reflect pure lactate dehydrogenase (LDH) activity since the signal also depends on the delivery of pyruvate, the rate of pyruvate uptake through the cell membrane monocarboxylate transporter 1 [31], and subsequently LDH activity and its substrate pool size [32]. Finally, due to the relative coarse spatial resolution, contributions from partial volume artifacts may also interfere with our results. In support of our findings, previous ex vivo studies have demonstrated increased cytosolic levels of both pyruvate and lactate in severe HF as a result of increased glycolysis and reduced pyruvate oxidation [11], [33], [34], [35]. It has been shown that levels of the mitochondrial pyruvate carrier, which is responsible for transporting pyruvate into the mitochondria, were reduced in severe HF, whereas levels of the cellular lactate exporter, monocarboxylate transporter-4, were increased [36]. As a result, more pyruvate was directed through LDH and converted to lactate. Such mechanisms may potentially account for at least part of the increased lactate signal that we observed in patients with severe HF. However, Murashige et al. recently found the failing heart to be a net consumer of lactate in a study using metabolomics on blood from artery and coronary sinus [37]. Similarly, animal positron emission tomography studies have shown a net extraction of lactate in the heart [38]. In tracer studies, Gertz et al. found evidence of regional myocardial lactate release despite global net extraction from the blood even in the absence of detectable ischemia [39], [40]. Hence, the fate of myocardial lactate remains disputable, and our findings suggest that hyperpolarized MRI may expand our understanding of lactate metabolism in HF.

### 4.2 Detection of bicarbonate as a measure of pyruvate oxidation in chronic heart failure

We found a significantly lower bicarbonate signal in patients with LVEF <30% than in other patients, supporting the previous pre-clinical findings of Fuetterer et al. [29]. The correlation between a poor contractility and a low bicarbonate signal was stronger in the IHD group alone. Previous studies on HP [1-^13^C]pyruvate CMR have established that the [^13^C]bicarbonate signal is trapped in the intracellular space during the timespan of the hyperpolarized ^13^C examination and therefore is a reliable marker of pyruvate oxidation [41]. Thus, findings by HP [1-^13^C]pyruvate CMR are in agreement with the general consensus on reduced mitochondrial pyruvate oxidation in patients with severe HF [11], [35], [42]. No previous studies have explored HP [1-^13^C]pyruvate MRI in HF in humans. However, Schroeder et al. reduced pyruvate oxidation in porcine DCM using HP [1-^13^C]pyruvate CMR [14]. Recently, reduced PDH flux in a porcine model of right ventricular HF was reported [43]. Previously, Rider et al. reported a reduced PDH activity in patients with type 2 diabetes, although without HF [16]. Insulin resistance may indirectly attenuate pyruvate oxidation by triggering the activity of PDH kinase and thus limiting the activity of the PDH complex [35], [44]. Apart from a decrease in PDH activity, other factors such as reduced mitochondrial pyruvate import [36] and a reduced number of mitochondria of smaller size, disorganized cristae, and decreased matrix density [33] may influence the detected bicarbonate signal. Thus, our findings show that hyperpolarized MRI is a feasible method to detect changes in pyruvate metabolism in patients with HF.

### 4.3 Baseline HP [1-^13^C]pyruvate CMR and LVEF improvement at follow-up

We found a strong correlation between the baseline lactate and bicarbonate ratios and LVEF improvement at follow-up in patients with IHD. These patients had no angina, and none of the patients were further revascularized in the follow-up period. Thus, our findings suggest that baseline HP [1-^13^C]pyruvate CMR may distinguish treatment responders in this subset of patients. However, in line with previous studies, in HF patients without IHD, we found no difference in metabolite ratios in responders versus non-responders and no correlation between baseline pyruvate metabolism and change in LVEF [45]. Thus, in patients with IHD and HF, HP [1-^13^C]pyruvate CMR following revascularization may assist in predicting treatment response.

## 5 Limitations

A limitation of this study was the small study population and that most patients with HF were in NYHA class II. Second, our healthy controls were significantly younger than the patients with HF. However, in the aging heart, mitochondrial function is known to be impaired [46]. Hence, increased glycolysis and PDH flux compensate for reduced fatty acid oxidation [47]. Therefore, the age difference in the present study may have weakened our ability to detect HF-induced reductions in PDH flux in the older patients with HF when compared with the younger healthy controls. Third, we used one short-axis mid-LV slice. Yet, this limitation can be overcome as recently demonstrated by Apps et al. who used three short-axis slices in HP [1-^13^C]pyruvate CMR [19]. Another limitation is the lack of LGE data in the ischemic patients. The intra-myocardial relationship between the regional metabolic patterns and LGE should be investigated in future studies. Finally, we used different FAs in patients and healthy controls. Differences in RF pulse sequence parameters can lead to systematic bias when comparing different studies and correction of sequence parameters is often needed [48]. We have aligned the results by presenting ratios of each metabolite to the TC, reducing the impact of this limitation. However, further work should investigate in detail the impact of the comparison of data from different acquisition protocols.

## 6 Conclusions

In this study, we demonstrated that HP [1-^13^C]pyruvate CMR can detect changes in pyruvate metabolism in patients with HF. Poor contractility, both globally and regionally, was associated with an increased lactate signal and reduced pyruvate oxidation. The encouraging finding of substantial inter-individual heterogeneity suggests that HP [1-^13^C]pyruvate CMR holds potential for phenotyping of patients with HF. The detection of a correlation between baseline pyruvate metabolism and treatment response in patients with IHD and HF suggests that HP [1-^13^C]pyruvate CMR may be useful in the clinical assessment of patients with HF.

## Clinical perspective

The present study suggests that HP [1-^13^C]pyruvate CMR may be useful in the clinical assessment of patients with HF and help in guiding treatment decisions in the future.

## Funding

This study was funded by grants from the 10.13039/501100004836Independent Research Fund Denmark (grant no. 9039-00157B), the 10.13039/100007405Danish Heart Foundation (grant no. 18-R124-A8429-22093), and the Department of Cardiology and the Centre of Clinical Research at the North Denmark Regional Hospital.

## Author contributions

S.H.J. obtained, analyzed, and interpreted data and drafted the first manuscript. E.S.S.H. obtained, interpreted, and analyzed data. N.B. and L.B. assisted in obtaining data and made major contributions to the writing process. R.S.T., P.S., C.L., and H.W. assisted in data interpretation and made major contributions to the writing process. All authors read and approved the final manuscript.

## Ethics approval and consent

The present study was conducted according to the Helsinki principles and approved by the National Committee on Health Research Ethics (2018-003533-15) and the Danish Medicines Agency (2019123690). Written informed consent was obtained from all patients before enrollment.

## Consent for publication

Not applicable.

## Declaration of competing interests

The authors declare that they have no known competing financial interests or personal relationships that could have appeared to influence the work reported in this paper.

## Data Availability

The authors declare that the data supporting the findings of this study are available within the paper. Raw data are available from the corresponding author upon reasonable request.

## Footnotes

Appendix A Supplementary data associated with this article can be found in the online version at doi:10.1016/j.jocmr.2024.101095.
