# Supplementary material for: Hyperpolarized [1-13C]pyruvate cardiovascular magnetic resonance imaging identifies metabolic phenotypes in patients with heart failure
Source: J Cardiovasc Magn Reson. 2024 Sep 11;26(2):101095. doi: 10.1016/j.jocmr.2024.101095 (PMC11635003; doi:10.1016/j.jocmr.2024.101095)
Supplement: Supplementary file 1 — Supplementary material [file mmc1.docx]

**SUPPLEMENTARY**

Figure 1. Correlation between Lac/Bic (A), Lac/TC (B) and Bic/TC (C) and age. о depicts IHD patients, ● depicts DCM patients.

Figure 2 Correlation between Lac/Bic (A), Lac/TC (B), and Bic/TC (C) and Body Mass Index. о depicts IHD patients, ● depicts DCM patients.

Figure 3 Correlation between Lac/Bic (A), Lac/TC (B), and Bic/TC (C) and LV mass Index. о depicts IHD patients, ● depicts DCM patients.

Figure 4 Correlation between Lac/Bic (A), Lac/TC (B), and Bic/TC (C) and nt-proBNP (ng/L). о depicts IHD patients, ● depicts DCM patients.

Figure 5. Plot of Lac/Bic (A), Lac/TC (B), and Bic/TC (C) and LVEF for both IHD patients: о, DCM patients: ●, and healthy controls: □.

Figure 6. Plot of Lac/Bic and HBA1C for all patients.

Figure 7. Correlation of circumferential strain and metabolite ratios for patients with heart failure (A-C), colour- and shape-coded for different patient cohorts and segments. о depicts IHD patients, ● depicts DCM patients. Green depicts anterior segment, blue antero-septal, brown antero-lateral, red inferior, yellow infero-septal and magenta infero-lateral.

Figure 9. Bullseye plot of the segmental relationship of circumferential strain and metabolite ratios for a IHD patient. Circumferential strain (A) Lac/Bic (B), Lac/TC (C), Bic/TC (D).

**
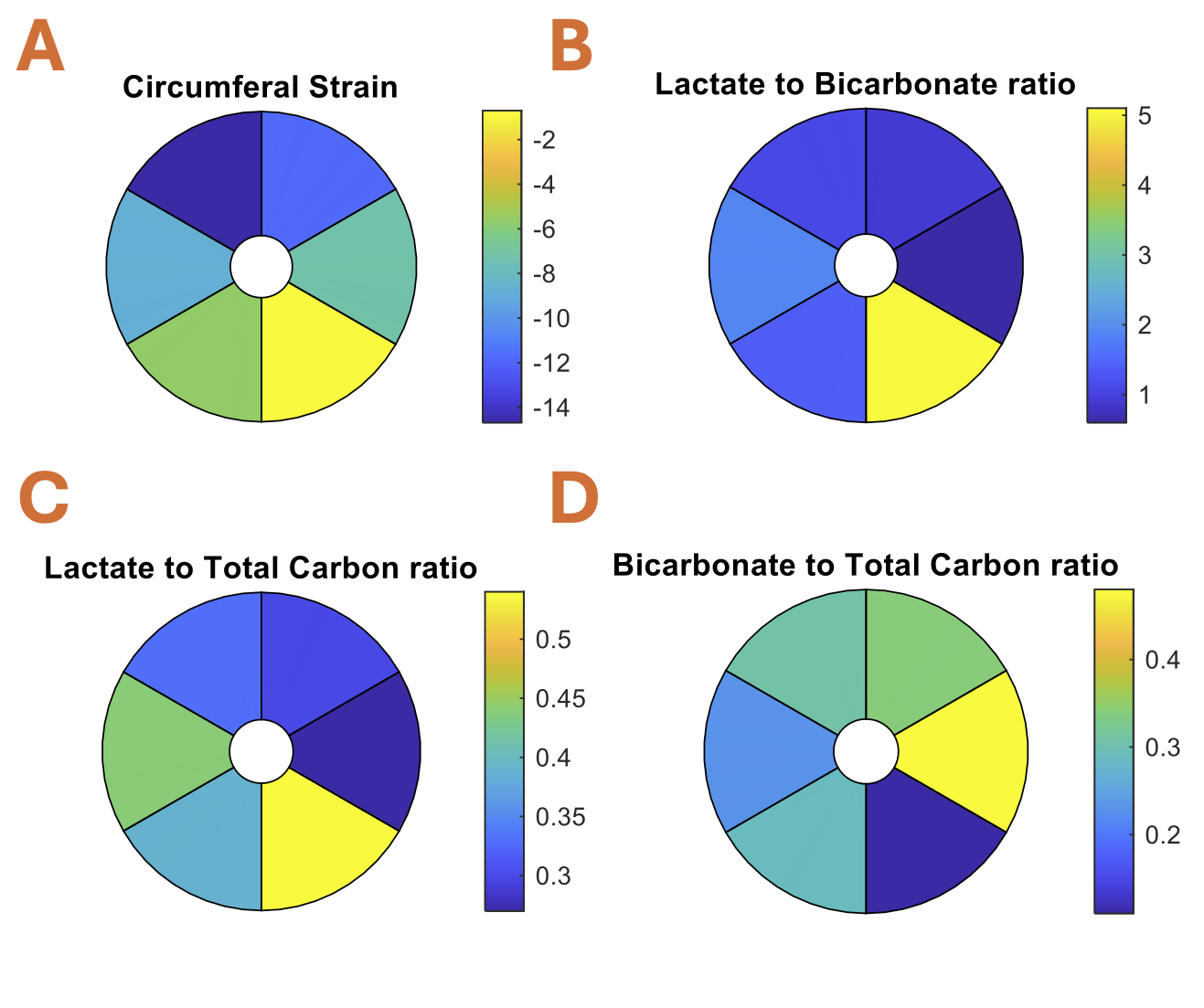
**
